# Trauma levels and coping strategies of health sciences students following the large-scale earthquakes in Türkiye

**DOI:** 10.1590/1980-220X-REEUSP-2026-0270en

**Published:** 2026-09-07

**Authors:** Gözde Yıldız Daş Geçim, Kübra Okuyucu

**Affiliations:** 1 Amasya University, Faculty of Health Sciences, Department of Public Health Nursing, Amasya, Türkiye.; 2 Amasya University, Faculty of Health Sciences, Department of Physiotherapy and Rehabilitation, Amasya, Türkiye.

**Keywords:** Coping Skills, Earthquake, Trauma and Stressor Related Disorders, Students, Türkiye, Habilidades de Enfrentamento, Terremotos, Transtornos Relacionados a Trauma e Fatores de Estresse, Estudantes, Turquia

## Abstract

**Objective:**

To investigate the trauma levels and associated coping strategies with earthquake stress experienced by health sciences students after the large-scale earthquakes that occurred in Türkiye in 2023.

**Method:**

This cross-sectional study was carried out using online surveys in April-July 2023 with 398 students living in Türkiye and studying the faculty of health sciences. Data were collected with a Participant Information Form, Post-Earthquake Trauma Level Determination Scale (PETLDS) and the Coping with Earthquake Stress Scale (CESS).

**Results:**

The students’ average PETLDS score was determined as 54.6 ± 16.81 (range: 20–100) indicating that the students were traumatised. The post-earthquake trauma levels were associated with the level of positive reappraisal coping (r = 0.186, p < 0.001). Compared to students from other departments, nursing students showed higher scores on the CESS subscales of religious coping (r = 0.110, p = 0.028), positive reappraisal (r = 0.224, p < 0.001), and seeking for social support (r = 0.137, p = 0.006), while reporting lower levels of trauma.

**Conclusion:**

Students exhibited high post-earthquake trauma levels, which were associated with several demographic and exposure-related factors. Compared with students from other health sciences departments, nursing students had lower trauma levels. They also reported higher use of positive reappraisal, religious coping, and seeking social support strategies.

## RESUMO

**Objetivo:**

Investigar os níveis de trauma e as estratégias de enfrentamento associadas ao estresse causado pelos terremotos vivenciados por estudantes das Ciências da Saúde após os terremotos de grande magnitude ocorridos na Turquia em 2023.

**Método:**

Este estudo transversal foi realizado por meio de questionários on-line entre abril e julho de 2023, com 398 estudantes residentes na Turquia e matriculados em faculdades de Ciências da Saúde. Os dados foram coletados utilizando um Formulário de Informações dos Participantes, a Escala de Determinação do Nível de Trauma Pós-Terremoto (PETLDS) e a Escala de Enfrentamento do Estresse Causado por Terremotos (CESS).

**Resultados:**

A pontuação média dos estudantes na PETLDS foi determinada como 54,6 ± 16,81 (variação: 20–100), indicando que os estudantes estavam traumatizados. Os níveis de trauma pós-terremoto estiveram associados ao nível de enfrentamento por reavaliação positiva (r = 0,186; p < 0,001). Em comparação com estudantes de outros departamentos das Ciências da Saúde, os estudantes de Enfermagem apresentaram escores mais elevados nas subescalas da CESS relacionadas ao enfrentamento religioso (r = 0,110; p = 0,028), à reavaliação positiva (r = 0,224; p < 0,001) e à busca por apoio social (r = 0,137; p = 0,006), enquanto relataram níveis mais baixos de trauma.

**Conclusão:**

Os estudantes apresentaram níveis elevados de trauma pós-terremoto, os quais estiveram associados a diversos fatores demográficos e relacionados à exposição. Em comparação com estudantes de outros departamentos das Ciências da Saúde, os estudantes de Enfermagem apresentaram níveis mais baixos de trauma. Além disso, relataram maior uso de estratégias de reavaliação positiva, enfrentamento religioso e busca de apoio social.

## INTRODUCTION

The February 2023 earthquakes in southeast Türkiye and Syria were among the most destructive natural disasters of the year. Three major quakes (Pazarcık Mw 7.7, Elbistan Mw 7.6, and Hatay Mw 6.4) caused widespread devastation across a large region, resulting in approximately 58,000 deaths, massive infrastructure damage, and an estimated US$104 billion in economic losses^(1,2)^. Following the 2023 earthquakes, many individuals in Türkiye experienced compounded hardships, including loss of life and property, homelessness, inadequate access to basic needs such as food, heating, clothing, and medical care, as well as large-scale displacement of over 216,347 people to other provinces^(1)^.

A further challenge concerned the continuity of education, as many students were forced to leave their cities due to loss of housing, and continued their studies in other provinces, often through dormitory placement and online learning. However, health sciences education relies heavily on clinical practice, making this transition particularly difficult. Disruptions of a similar nature during the COVID-19 pandemic had already been associated with increased anxiety, largely due to limitations in clinical training^(3)^, while inadequate internet access further contributed to elevated stress levels^(4)^. When combined with disaster-related trauma, grief, and uncertainty regarding academic progression, these factors were associated with increased psychological distresss.

Earthquakes are profound traumatic stressors that impose a substantial psychological burden on affected populations, with post-traumatic stress responses shaped by both the intensity and duration of exposure^(5,6,7)^. Beyond their immediate effects, earthquakes disrupt academic functioning while simultaneously challenging psychological resilience and coping capacity. Therefore, a comprehensive understanding of coping processes in this population is essential not only for protecting individual mental health but also for preparing future healthcare professionals for roles in disaster and crisis settings. From a psychosocial nursing perspective, these responses provide a critical framework for identifying support needs and strengthening adaptive coping mechanisms.

International research consistently shows that adults and university students exposed to natural disasters face increased psychological distress, including post-traumatic stress symptoms, anxiety, and depression^(6,8,9)^. Although these studies have substantially improved our understanding of disaster-related psychological outcomes, evidence regarding the concurrent assessment of trauma and coping among university students is still insufficient. Despite the growing disaster literature, research on post-earthquake trauma and coping strategies among health sciences students in Türkiye remains limited^(10,11)^. This gap is critical, as these students represent both directly affected individuals and future healthcare providers in disaster settings, where dual exposure may be associated with higher distress and coping patterns. Given the possibility of future earthquakes in Türkiye and the risk of student re-traumatization, psychological effects may persist over time, contributing to sustained trauma, fear, and anxiety, as well as impairments in social functioning and support systems. Accordingly, a concurrent examination of trauma and coping is essential to capture students’ psychosocial status and to identify both vulnerabilities and protective factors that may inform targeted interventions to enhance resilience and disaster preparedness.

This study is grounded in Lazarus and Folkman’s Transactional Model of Stress and Coping, which provides a comprehensive framework for understanding how individuals respond to stressful life events^(12)^. According to this model, psychological outcomes emerge from the dynamic interaction between stressors, cognitive appraisals, and coping strategies. It also highlights psychosocial dimensions by emphasizing the role of environmental and social resources in the stress and coping process. In this context, the model provides a useful framework for examining the relationship between earthquake-related stressors, coping strategies, and trauma levels among health sciences students. Given that Türkiye is located in an active seismic zone and that earthquake risk remains a major public health concern, understanding post-earthquake psychological adjustment among health sciences students is of considerable importance. These students are not only directly affected individuals but also future healthcare providers expected to play key roles in disaster response. Accordingly, this study aimed to investigate the trauma levels and associated coping strategies with earthquake stress experienced by health sciences students after the large-scale earthquakes that occurred in Türkiye in 2023.

### Research Questions

(1) What are the post-earthquake trauma levels of health sciences students?
(2) What coping strategies do health sciences students use?
(3) Is there a relationship between trauma levels and coping strategies?
(4) Which demographic and experiential factors are associated with trauma levels and coping strategies?

## METHOD

### Research Type

This is a cross-sectional study.

### Population and Research Sample

This study was conducted using a self-reported online survey between 10 April 2023 and 25 July 2023. The target population consisted of students enrolled in faculties of health sciences at universities in Türkiye. A non-probability sampling strategy was employed, including convenience and snowball sampling methods. A publicly available statistical software (OpenEpi v.3) was used to estimate the minimum required sample size, which was calculated as 385 participants based on a significance level of 0.05 and a 95% confidence interval^(13)^. A total of 398 students participated in the study, exceeding the minimum required sample size and thereby ensuring sufficient statistical power for the analyses. Participants were recruited through lecturers and student social media groups. Because convenience and snowball sampling were used and the survey link was disseminated through multiple channels, the total number of students who received the invitation could not be determined; therefore, a response rate could not be calculated.

### Selection Criteria

The inclusion criteria were: (1) being 18 years of age or older, (2) being a student in a health sciences program (eligibility was determined using a self-report screening question asking whether participants were enrolled in a health sciences program), (3) voluntarily agreeing to participate, and (4) having internet access. Individuals who reported severe physical or mental health conditions that could prevent survey completion were not included. The exclusion criteria were: (1) self-reported hearing or visual impairments that could interfere with participation and (2) withdrawal from the study at any stage.

### Data Collection

The survey included questions about participant characteristics as well as two pre-developed and validated scales (Postearthquake Trauma Level Determination Scale (PETLDS)^(14)^and Coping with Earthquake Stress Scale (CESS)^(15)^. Once Amasya University Ethics Committee provided ethical clearance, all students enrolled in undergraduate health sciences education at all universities in Türkiye were invited to participate in the study and complete the anonymised online survey designed using Google Forms. The survey link was initially distributed to lecturers and student representatives, who were asked to forward it to eligible students through institutional communication channels and student social media groups. Participants were also encouraged to share the survey with other eligible health sciences students. Google Forms settings were configured to restrict multiple submissions from the same account.

The students who accepted to participate in the study saw the Information Sheet and Consent Form as the first page when they clicked on the link, and after being asked to thoroughly read the information sheet and consent form, they were asked to electronically sign their approval to the form, after which they had online access to the data collection forms in the form of a survey. The online survey was designed to ensure participant anonymity by not collecting any personally identifiable information. Data were stored on a secure, password-protected platform accessible only to the researchers. All responses were confidential, and participation was voluntary. These measures complied with ethical guidelines for research involving sensitive trauma-related data to protect participants’ privacy and encourage correct responses. The survey link was disseminated to students through course lecturers forwarding the survey link to their students via email. The survey was publicised on health sciences students’ social media (Facebook, Instagram and WhatsApp) groups. Regular reminders were sent to the lecturers, and regular posts were shared in popular social media groups to encourage students to fill the survey every two weeks. The average completion time for the survey was approximately 10–15 minutes.

### Data Collection Tools

***Participant Information Form (PIF):*** This form was created by the researchers in accordance with the literature^(14,16)^. The participant characteristics questions included age, gender, health sciences department, city of residence, experience of the February 6 earthquake, the effect of the earthquake on physical and mental health, and the impact of the earthquake on education.

***Post-Earthquake Trauma Level Determination Scale (PETLDS):*** The PETLDS was developed in the Turkish language and validated in 2013 by Tanhan and Kayri^(14)^ to measure the trauma level after an earthquake. It consists of 20 items grouped into five sub-scales: (1) cognitive structure, (2) behavioural problems, (3) affective behaviours, (4) emotional limitation, and (5) sleeping problems. Each question is measured using the five-point Likert type scale (from 1 to 5, 1 representing not at all and 5 representing always), with total scores ranging from 20 to 100. A higher score indicated a higher level of trauma after an earthquake. A score of 52.385 ± 5.051 according to the scale indicates a threshold value at which individuals are traumatized^(14)^. Values above and below this threshold value indicates low and high levels of traumatization respectively. The PETLDS is a Turkish instrument known for its strong scientific validity, with a Cronbach’s alpha of 0.87, which demonstrates good internal consistency^(14)^.

***Coping with Earthquake Stress Scale (CESS):*** The CESS was developed in Turkish language and validated by Yondem and Altay^(15)^. The 16-item scale measures emotions, thoughts and behaviours used to cope with earthquake stress. It consists of three subscales of (1) religious coping (questions 2, 3, 9, 10 and 11), (2) positive reappraisal (questions 7, 12, 13, 14, 15, and 16), and seeking for (3) social support (questions 1, 3, 4, 6, and 7). Each question is measured using the four-point Likert scale (from 1 to 4, 1 representing not at all, 4 representing always) and scored 1 to 4. The scale does not reveal a total score, as each sub-scale is scored on its own. The total scores ranged from 5 to 20 for religious coping, 6 to 24 for positive reappraisal and 5 to 20 for seeking for social support. Higher scores indicate more use of this coping strategy^(15)^. The Cronbach’s alpha coefficients for the CESS were found to be α = 0.85 for Religious Coping, α = 0.69 for Positive Reappraisal, and α = 0.74 for Seeking Social Support^(15)^.

### Ethical Aspects

All participants were informed of the study details and agreed to participate in the research. The study was approved by the Amasya University Ethics Committee (Date: 10.04.2023; Approval No: E-76988455-050.01.04-125195). Ethical approval was obtained prior to data collection. All students registered in undergraduate health sciences education programs at universities in Türkiye were invited to participate. The first page of the online survey (Google Forms) included an Information Sheet and an electronic Consent Form. Participants were required to read the information sheet and provide electronic informed consent before accessing the questionnaire. Those who provided consent participated in the study and completed the survey. The study was conducted in accordance with the Declaration of Helsinki. Artificial intelligence was not used in any part of this study.

### Data Analysis

The data were entered and analysed by using the program SPSS for Windows 24.0. (IBM Inc, Armonk, NY, USA) statistics package program. Minimum, maximum, mean, standard deviation, frequency and percentage were used to report the data. Descriptive statistics (mean, standard deviation [SD] or number, frequency) were used to report the data. The Kolmogorov Smirnov test was used to evaluate the normality of data prior to test selection, which revealed that the data were normally distributed. Pearson’s Correlation Coefficient (for relationships between PETLDS, CESS, and age) and Spearman’s Correlation Coefficient (for relationships with other categorical variables) were used to examine relationships between trauma levels and coping strategies. These correlation analyses are appropriate methods for investigating relationships between variables. Rho values were reported to show the strength of the association. Multiple linear regression analyses were conducted to identify factors that potentially predict trauma levels and coping strategies. Since the study relied on self-report survey data, Harman’s single-factor test was conducted to assess the risk of common method variance (CMV). An unrotated exploratory factor analysis (EFA) was performed using all items from the measurement scales. All participants completed all questions; there was no missing data. The results were evaluated at the 95% confidence interval and the significance level of p < 0.05.

## RESULTS

A total of 398 students completed the survey. The mean age of the participants was 20.9 ± 1.7 years (range: 18–30 years). A majority of the students were female (82%) and studying nursing (55%). One third of the students (33%) reported experiencing the earthquake. Many of the students (39%) were receiving distance learning. Half of the students (50%) reported that the earthquake had a bad effect on their lives (Table 1).

**Table 1:** Characteristics of the participants (N = 398) – TR, Türkiye, 2023.

Variables	Number (%)
**Gender**	
Female	325 (82)
Male	73 (18)
**Department**	
Nursing	218 (55)
Midwifery	81 (20)
Nutrition and dietetics	53 (13)
Physiotherapy	46 (12)
**Experience of the earthquake**	
Yes	133 (33)
No	265 (67)
**Existence of any health problems due to the earthquake**	
Yes	56 (14)
No	342 (86)
**Physical health status after the earthquake**	
Very good	6 (2)
Good	24 (6)
Acceptable	233 (59)
Poor	101 (25)
Very poor	34 (8)
**Psychological health status after the earthquake**	
Very good	0
Good	1 (1)
Acceptable	26 (6)
Poor	184 (46)
Very poor	187 (47)
**Current status of education**	
Distance learning	156 (39)
Face to face	98 (25)
Hybrid	144 (36)
**Expected status of education**	
Distance learning	113 (29)
Face to face	163 (41)
Hybrid	71 (17)
Paused	51 (13)
**Impact of the earthquake on life**	
Very positive	3 (1)
Positive	3 (1)
Neither positive nor negative	99 (25)
Negative	199 (50)
Very negative	94 (23)
**Frequency of following earthquake news**	
Always	109 (27)
Very often	204 (51)
Sometimes	63 (16)
Rarely	15 (4)
Never	7 (2)

### Post-Earthquake Trauma Levels and Coping Strategies

The mean PETLDS score was 54.6 ± 16.81, indicating that the students were traumatised. Among the departments, students studying nursing were less traumatised than students from other departments. Similarly, the coping levels of earthquake stress of nursing students were the highest (Table 2).

**Table 2:** Mean value of trauma levels and strategies for coping with earthquake stress by department – TR, Türkiye, 2023.

Department	N
		Post-earthquake trauma level (range: 20–100)		Strategies for coping with earthquake stress					
				Religious coping (range: 5–20)		Positive reappraisal (range: 6–24)		Seeking for social support (range: 5–20)	
		mean	SD/SE	mean	SD/SE	mean	SD/SE	mean	SD/SE
Nursing	218	51.6	1.12	14.8	0.23	17.1	0.22	13.9	0.13
Midwifery	81	56.7	1.8	13.9	0.38	16.3	0.38	12.9	0.23
Nutrition and dietetics	53	61.9	1.97	13.5	0.51	15.1	0.51	12.3	0.3
Physiotherapy	46	56.6	2.69	13.6	0.54	15.3	0.46	12.6	0.33
Total	398	54.6	16.8	14.5	3.55	16.5	3.49	13.1	2.12

N: number; SD: standard deviation; SE: standard error.

### Relationship Between PETLDS and CESS Factors

The results of the correlation analysis between PETLDS and coping levels revealed that the post-earthquake trauma levels were associated with the level of positive reappraisal coping (r = −0.186, p < 0.001). As the post-earthquake trauma levels of students increased, levels of coping with earthquake stress by positive appraisal decreased (Table 3).

**Table 3:** Statistically significant associations between post-earthquake trauma levels, coping strategies with earthquake stress and other factors – TR, Türkiye, 2023.

	1	2	3	4	5	6	7	8	9	10
Gender	–									
Department	.120^*^	–								
Experience of the earthquake	.091	.058	–							
Existence of any health problems due to the earthquake	.107^*^	.066	.188^***^	–						
Effect of the earthquake on education	.062	.015	.114^*^	.150^***^	–					
Frequency of following earthquake news	.086	.086	.030	.047	.136^***^	–				
PETL	.160^***^	.197^***^	.141^***^	.141^***^	.199^***^	.208^***^	–			
Religious coping	.059	.110^*^	.038	.037	.163^**^	.062	.098	–		
Positive reappraisal	.087	.224^***^	.018	.016	.110^*^	.018	.186^***^	.439^***^	–	
Seeking for social support	.064	.134^**^	.054	.004	.049	.137^**^	.045	.278^***^	.464^***^	–

The table presents the r/rho values.

*p < 0.05;

**p < 0.01;

***p < 0.001.

PETL: Post-earthquake Trauma Level.

1. Gender; 2. Department; 3. Experience of the earthquake; 4. Existence of any health problems due to the earthquake; 5. Effect of the earthquake on education; 6. Frequency of following earthquake news; 7. PETL; 8. Religious coping; 9. Positive reappraisal; 10. Seeking for social support.

### Factors Associated With PETLDS and CESS

PETLDS scores showed significant associations with several demographic and experiential factors. Female students reported higher trauma levels than male students. Departmental differences were also evident: nursing students had lower PETLDS scores than students from other health sciences disciplines (midwifery, nutrition and dietetics, physiotherapy). Moreover, students who directly experienced the earthquake, either by being present in the affected area or through physical or emotional exposure, had significantly higher PETLDS scores than those who were not directly affected. Similarly, students who experienced earthquake-related health problems (such as injury or illness) also reported elevated trauma levels. There was also a positive association between PETLDS scores and students’ perception that the earthquake might impact their education. Those who felt that the disaster disrupted their academic life were more likely to report higher trauma levels. Additionally, a higher frequency of following earthquake-related news was associated with greater trauma levels (Table 3).

Religious coping with earthquake stress was associated with the study department (nursing) and the effect of the earthquake on education. Nursing students had higher levels of religious coping (r = 0.110, p = 0.028). There was a weak negative correlation between religious coping and the possible effect of the earthquake on education (r = −0.163, p = 0.001); those who thought the earthquake would affect education had lower levels of religious coping. Similarly, positive reappraisal coping with earthquake stress was also associated with the study department and the effect of the earthquake on education. Nursing students had higher levels of positive reappraisal (r = 0.224, p < 0.001), and there was a weak negative correlation between positive reappraisal coping and the effect of the earthquake on education (r = −0.110, p = 0.028) (Table 3).

Seeking for social support coping levels were found to be associated with the students’ departmental affiliation, with nursing students performing at higher levels than students in other departments (r = 0.134, p = 0.007). It was also associated with the frequency of following earthquake news. Those who followed the news more often had lower levels of seeking for social support to cope with earthquake stress (r = −0.137, p = 0.006) (Table 3).

A multiple linear regression analysis was conducted to examine whether demographic and earthquake-related variables predicted trauma levels. The independent variables were age, gender, department, year of study, experience of the earthquake, frequency of following earthquake news and effect of the earthquake on education. Among the predictors, gender was a significant predictor, with females reporting higher trauma levels than males (β = -0.153, p = .002). Department was positively associated with trauma scores (β = 0.156, p = .001), indicating differences among academic programs. Year of study was a negative predictor (β = -0.128, p = .025), suggesting that students in more advanced years experienced lower trauma levels. Experiencing an earthquake was also associated with higher trauma levels (β = -0.152, p = .002), as was the frequency of following earthquake-related news (β = -0.177, p <.001). Age approached significance (β = 0.106, p =.060) but was not statistically significant, and the perceived effect of the earthquake on education was not a significant predictor (β = -0.013, p = .785).

A multiple regression analysis was conducted to examine whether demographic and earthquake-related variables predicted coping strategies. Among the predictors, department was a statistically significant predictor for religious coping (β = -0.133, p = .008), positive reappraisal (β = -0.222, p < .001), and seeking social support (β = -0.157, p = .002), indicating that students from the nursing department reported higher scores on these coping strategies compared to students from other departments. Additionally, frequency of following earthquake-related news significantly predicted seeking social support (β = -0.144, p = .004), suggesting that students who followed the news more frequently reported lower levels of seeking social support.

## DISCUSSION

The February 2023 earthquakes in Türkiye, described as the “*disaster of the century*,” were associated with substantial physical, psychological, and psychosocial challenges. This study aimed to investigate the trauma levels and associated coping strategies with earthquake stress experienced by health sciences students after the large-scale earthquakes that occurred in Türkiye in 2023.

### Post-Earthquake Trauma

Natural disasters such as earthquakes are one of the important events that cause trauma^(16)^ and they have a significant impact on the mental health of survivors^(8,17)^. Post-earthquake trauma levels are shown to be associated with a variety of mental and physical health problems in the vast majority of the general population^(16,18)^. In this study, the mean PETLDS score was found as 54.6 ± 16.81. Tanhan and Kayri^(14)^ state that scores above the cut-off value of 52.385 ± 5.051 indicate that the students are traumatized. This value was found to be 23.12 ± 15.03 in the study by Kardas and Tanhan^(16)^ with university students who had experienced the Van earthquake in Türkiye. In a study by Çakır et al.^(19)^, which examined trauma levels and earthquake stress coping strategies among university students who exercised and those who did not after an earthquake, the value was found to be 70.94. These differences may be associated with variations in the severity and scope of the disasters, the level of direct exposure, and the timing of data collection relative to the event. Specifically, differences in sample characteristics such as age, gender, socioeconomic status, and whether participants were directly or indirectly exposed to the earthquake may have influenced reported trauma levels. Additionally, the type of measurement tools and data collection methods used could have affected the outcomes. Cultural context may also have played an important role, as collective trauma processing, social support systems, and access to psychosocial services vary across regions and communities.

In a study by Yokoyama et al.^(20)^, which examined mental health following the 11 March 2011 East Japan earthquake and tsunami, mental health problems were found to be highly prevalent among survivors. Similarly, a systematic review and meta-analysis on post-traumatic stress disorder highlighted the necessity for local governments to implement effective psychological interventions for individuals affected by earthquakes^(17)^. Research also indicates that individuals’ responses to trauma are associated with their coping mechanisms^(6,19)^. Therefore, strengthening coping capacity requires the immediate and comprehensive addressing of basic needs such as housing, nutrition, education, and security following disasters. This is especially crucial for university students whose education is disrupted-not only by a sudden shift to remote learning but also by the inability to complete essential hands-on clinical or laboratory training. Implementing culturally sensitive interventions that respect societal values and collective coping styles can provide targeted psychological support and help restore both academic continuity and overall well-being.

### Factors Associated with Students’ PETLDS and CESS

In the study, higher levels of post-earthquake trauma were observed among female students, those who directly experienced the earthquake, those reporting health problems, those who perceived their education as affected, and those frequently exposed to earthquake-related news. These associations may reflect the co-occurrence of multiple stressors such as loss of loved ones, property damage, and disruption of daily life and education. Ongoing media exposure may have been related to heightened emotional arousal and sustained psychological distress over time, potentially intensifying traumatic memories. In addition, the abrupt shift to online education may have been associated with increased academic disruption, reduced social support, and heightened uncertainty. Notably, nursing students reported lower trauma levels and stronger coping skills across all sub-dimensions of the CESS. One possible explanation is that nursing students may benefit from early clinical exposure and training related to stress management and disaster preparedness. However, these factors were not directly measured in this study and therefore should be interpreted cautiously. Furthermore, these differences may also be influenced by self-selection into the nursing program, as well as potential variations in demographic characteristics such as gender, distribution, and age, and prior exposure to healthcare-related or stressful experiences across academic disciplines. However, most of the observed associations were weak in magnitude. Although statistically significant, these findings may have limited practical significance and should be interpreted with caution. Additional contextual factors such as institutional support systems, socioeconomic conditions, and access to mental health services may also be relevant in explaining differences across student groups.

In accordance with the present results, previous studies in literature have demonstrated that the common risk factors for post-earthquake are female gender^(19,20)^, older age^(21)^, low education level and socioeconomic status^(22)^, exposure to earthquake, previous psychiatric illness^(23)^, post-earthquake displacement, poor social networks, and financial losses^(24)^.

### Associations Between Students’ PETLDS on CESS

In the study, there was a statistically significant negative relationship between post-earthquake trauma levels and the students’ ability to cope with earthquake stress with positive reappraisal. Positive reappraisal, a cognitive process that involves reframing a stressful event to find personal growth or meaning, is linked to greater psychological well-being and is considered a problem-focused coping mechanism^(15)^. It is central to cognitive-behavioural therapies for depression and stress management, highlighting its role in adaptive coping and resilience^(25,26)^. The observed negative association suggest that students reporting greater use of positive reappraisal also tended to report lower trauma levels. However, the cross-sectional nature of the study does not allow conclusions regarding whether positive reappraisal reduces trauma symptoms or whether lower trauma facilitates the use of positive reappraisal. This finding also aligns with Lazarus and Folkman’s Transactional Model of Stress and Coping, where rephrasing stressors is an emotion-focused coping technique that builds resilience and reduces perceived threat^(12)^. Positive reappraisal may help individuals feel more in control and cope better, which can protect them from the psychological impact of traumatic events. Nursing students in this study reported higher levels of positive reappraisal, along with lower trauma scores, further supporting the theory that stronger internal coping mechanisms are associated with reduced trauma impact. In contrast, higher trauma levels may be associated with reduced use of such adaptive cognitive strategies, which are essential for psychological resilience. These findings suggest that psychosocial interventions and training programs aimed at strengthening adaptive coping strategies warrant further investigation, although their effectiveness cannot be inferred from the present study.

In addition, a positive association was found between students’ perception that the earthquake affected their educational life and both positive reappraisal and religious coping. This associateion may indicate that students experiencing greater educational disruption were more likely to report using positive reapprahisal and religious coping strategies. In some studies, it has been found that individuals resort to positive religious coping methods after earthquakes^(19,27)^. Religious coping, as an emotion-focused strategy, can provide meaning and comfort and may facilitate positive reappraisal by helping individuals reinterpret adverse events in a more constructive way. Positive reappraisal, in turn, reflects a cognitive effort to reconstruct stressful experiences in a way that enhances perceived control and emotional stability. Some studies indicate that religious coping is a common and effective way of managing stress during traumatic events such as earthquakes; likewise, viewing adversity within a spiritual framework and seeking strength through faith has been shown to alleviate distress and support psychological well-being^(15,25)^. In culturally religious contexts such as Türkiye, disasters are often interpreted within a spiritual framework, and prayer and other religious practices serve as key coping resources that provide comfort and resilience^(8,28)^.

Spirituality and religion play a significant role in coping with stress and adaptation^(29,30)^, and these values have been shown to enhance post-traumatic psychological well-being^(31)^. The present study’s finding that religious coping was the most frequently used strategy among students is consistent with this evidence and underscores the interaction between individual coping processes and broader cultural value systems^(32)^. In addition, educational environments, which are central to students’ social and psychological development, may further shape the adoption of these coping strategies by influencing how students manage academic responsibilities alongside the emotional burden of disaster exposure. Furthermore, the findings related to seeking social support indicate that coping responses to disaster-related stress are shaped by both academic context and information exposure. Specifically, students from the nursing department reported higher scores on seeking social support than students from other departments, suggesting that disciplinary differences may be associated with variations in support-seeking behaviours. In this context, seeking social support reflects an active coping effort directed toward obtaining emotional and instrumental assistance following a disaster. This interpretation is partially supported by prior research demonstrating that higher perceived social support is associated with more effective coping among nursing students^(33)^, highlighting the functional role of interpersonal resources in stress regulation. In contrast, more frequent exposure to earthquake-related news was associated with lower scores on this subscale. This association may reflect reduced support-seeking among students reporting greater media exposure, although the direction of this relationship cannot be determined. Overall, these findings emphasize that effective post-disaster interventions for students should adopt an integrated framework that combines evidence-based psychological approaches with culturally and spiritually sensitive perspectives to strengthen resilience and support long-term recovery.

### Study Strengths and Limitations

This study has several strengths and limitations. To the best of our knowledge, it is the first study to examine postearthquake trauma levels and coping strategies among health sciences students in Türkiye following the 2023 earthquakes. It provides preliminary evidence that may serve as a foundation for future research on trauma responses and coping mechanisms in disaster-exposed populations, particularly within health sciences education. Although similar studies exist in other university populations, evidence focusing specifically on health sciences students remains limited, which strengthens the contribution of this study. The findings also highlight the importance of integrating stress management and coping strategy training into health sciences curricula to better prepare students for future disaster-related psychological challenges. Overall, the results underscore the need for resilience-focused and trauma-informed education in health sciences, with important implications for nursing and health education curricula.

Despite these strengths, several limitations should be acknowledged. First, a non-probability sampling approach (convenience and snowball sampling via online platforms and university networks) was used, which may have introduced selection bias, and limits the representativeness of the sample. Consequently, the findings should be interpreted with caution and may not be generalisable to all health sciences students in Türkiye. Although the sample was adequate for statistical analyses, participants were recruited from multiple universities without systematically recording institutional distribution, and the sample was imbalanced in terms of gender and field of study, which may have affected representativeness.

Second, the cross-sectional design precludes conclusions about causal relationships. Therefore, the associations observed between trauma levels, coping strategies, and participant characteristics should not be interpreted as evidence of cause- and-effect relationships. In addition, a proportion of the participants had experienced the earthquake indirectly, which may have influenced the reported trauma levels and should be considered when interpreting the findings. The study also did not assess potentially important variables, including previous psychiatric history, previous traumatic experiences, self-efficacy, perceived social support, and sociocultural context, all of which may have influenced trauma responses and coping strategies. Finally, the use of self-reported measures may have introduced response and social desirability bias. Future longitudinal studies using probability-based sampling methods are needed to confirm these findings and to provide a more comprehensive understanding of trauma levels and coping processes among health sciences students.

## CONCLUSION

This study provides important insights into university students’ trauma levels and coping strategies following the earthquakes that occurred on 6 and 20 February 2023. The findings indicate that overall post-earthquake trauma levels among health sciences students were high. Trauma levels were significantly associated with several factors, including gender, academic department, perceived disruption of academic life, direct exposure to the earthquake, earthquake-related health problems, and the frequency of following earthquake-related news. In addition, nursing students demonstrated lower levels of trauma and stronger coping abilities compared to students from other departments.

Further analysis showed that nursing students reported greater use of religious coping, positive reappraisal, and seeking social support, while also experiencing lower levels of trauma. These findings are consistent with Lazarus and Folkman’s Transactional Model of Stress and Coping, supporting the role of cognitive appraisal processes and coping strategies in shaping individuals’ psychological responses to traumatic events.

Overall, the results highlight the need for comprehensive emotional, social, and psychological support systems for health sciences students, particularly given their future roles as frontline healthcare professionals. These results indicate that adaptive coping strategies such as positive reappraisal, religious coping, and social support utilization, as well as the inclusion of coping skills and disaster preparedness training in health sciences curricula, may support students’ psychological adjustment following disasters. The study also underscores the importance of further research to deepen understanding of post-disaster trauma processes and coping mechanisms.

## DATA AVAILABILITY

The entire dataset supporting the results of this study was published in the article itself.
