# Supplementary material for: Trauma levels and coping strategies of health sciences students following the large-scale earthquakes in Türkiye
Source: Rev Esc Enferm USP. 2026 Sep 7;60:e20260270. doi: 10.1590/1980-220X-REEUSP-2026-0270en (PMC13561536; doi:10.1590/1980-220X-REEUSP-2026-0270en)
Supplement: Translation

# Translation: Niveles de trauma y estrategias de afrontamiento de estudiantes de ciencias de la salud tras los terremotos de gran magnitud en Turquia

**DOI:** 10.1590/1980-220X-REEUSP-2026-0270es

**Authors:** Gözde Yıldız Daş Geçim, Kübra Okuyucu

**Affiliations:** 1 Amasya University, Faculty of Health Sciences, Department of Public Health Nursing, Amasya, Türkiye.; 2 Amasya University, Faculty of Health Sciences, Department of Physiotherapy and Rehabilitation, Amasya, Türkiye.

**Keywords:** Estrategias de Afrontamiento, Terremotos, Trastornos Relacionados con Traumatismos y Factores de Estrés, Estudiantes, Turquía

## Abstract

**Objetivo:**

Investigar los niveles de trauma y las estrategias de afrontamiento asociadas al estrés provocado por los terremotos experimentados por estudiantes de Ciencias de la Salud después de los terremotos de gran magnitud ocurridos en Turquía en 2023.

**Método:**

Este estudio transversal se llevó a cabo mediante encuestas en línea entre abril y julio de 2023 con 398 estudiantes residentes en Türkiye y matriculados en facultades de Ciencias de la Salud. Los datos fueron recopilados mediante un Formulario de Información del Participante, la Escala de Determinación del Nivel de Trauma Posterior al Terremoto (PETLDS) y la Escala de Afrontamiento del Estrés por Terremotos (CESS).

**Resultados:**

La puntuación media de los estudiantes en la PETLDS fue determinada como 54,6 ± 16,81 (rango: 20–100), lo que indicó que los estudiantes estaban traumatizados. Los niveles de trauma posteriores al terremoto se asociaron con el nivel de afrontamiento mediante reevaluación positiva (r = 0,186; p < 0,001). En comparación con los estudiantes de otros departamentos de Ciencias de la Salud, los estudiantes de Enfermería obtuvieron puntuaciones más elevadas en las subescalas de la CESS relacionadas con el afrontamiento religioso (r = 0,110; p = 0,028), la reevaluación positiva (r = 0,224; p < 0,001) y la búsqueda de apoyo social (r = 0,137; p = 0,006), además de presentar menores niveles de trauma.

**Conclusión:**

Los estudiantes presentaron altos niveles de trauma posterior al terremoto, los cuales estuvieron asociados con diversos factores demográficos y relacionados con la exposición al desastre. En comparación con los estudiantes de otros departamentos de Ciencias de la Salud, los estudiantes de Enfermería mostraron menores niveles de trauma. Asimismo, informaron un mayor uso de estrategias de reevaluación positiva, afrontamiento religioso y búsqueda de apoyo social.

## INTRODUCCIÓN

Los terremotos de febrero de 2023 en el sureste de Turquía y Siria se contaron entre los desastres naturales más destructivos del año. Tres sismos importantes (Pazarcık Mw 7,7, Elbistan Mw 7,6 y Hatay Mw 6,4) causaron una devastación generalizada en una amplia región, lo que provocó aproximadamente 58 000 muertes, daños masivos a la infraestructura y pérdidas económicas estimadas en 104 mil millones de dólares de EE. UU^(1,2)^. Tras los sismos de 2023, muchas personas en Turquía sufrieron dificultades agravadas, entre ellas la pérdida de vidas y bienes, la población en situación de calle, el acceso insuficiente a necesidades básicas como alimentos, calefacción, ropa y atención médica, así como el desplazamiento a gran escala de más de 216 347 personas a otras provincias^(1)^.

Otro desafío se relacionó con la continuidad de la educación, ya que muchos estudiantes se vieron obligados a abandonar sus ciudades debido a la pérdida de su vivienda y continuaron sus estudios en otras provincias, a menudo mediante el alojamiento en residencias estudiantiles y el aprendizaje en línea. Sin embargo, la educación en ciencias de la salud depende en gran medida de la práctica clínica, lo que hace que esta transición sea particularmente difícil. Las interrupciones de naturaleza similar durante la pandemia de COVID-19 ya se habían asociado con un aumento de la ansiedad, en gran parte debido a las limitaciones en la capacitación clínica^(3)^, mientras que el acceso inadecuado a Internet contribuyó aún más a elevar los niveles de estrés^(4)^. Al combinarse con el trauma relacionado con el desastre, el duelo y la incertidumbre respecto a la progresión académica, estos factores se asociaron con un aumento del malestar psicológico.

Los terremotos son factores estresantes traumáticos profundos que imponen una carga psicológica sustancial a las poblaciones afectadas, y las respuestas de estrés postraumático se ven determinadas tanto por la intensidad como por la duración de la exposición^(5,6,7)^. Más allá de sus efectos inmediatos, los terremotos interrumpen el funcionamiento académico y, al mismo tiempo, ponen a prueba la resiliencia psicológica y la capacidad de afrontamiento (coping). Por lo tanto, una comprensión integral de los procesos de afrontamiento en esta población es esencial no solo para proteger la salud mental individual, sino también para preparar a los futuros profesionales de la salud para desempeñar funciones en situaciones de desastre y crisis. Desde una perspectiva de enfermería psicosocial, estas respuestas proporcionan un marco fundamental para identificar las necesidades de apoyo y fortalecer los mecanismos de afrontamiento adaptativos.

Las investigaciones internacionales muestran de manera consistente que los adultos y los estudiantes universitarios expuestos a desastres naturales enfrentan un mayor malestar psicológico, incluyendo síntomas de estrés postraumático, ansiedad y depresión^(6,8,9)^. Aunque estos estudios han mejorado sustancialmente nuestra comprensión de los resultados psicológicos relacionados con los desastres, la evidencia sobre la evaluación simultánea del trauma y el afrontamiento entre los estudiantes universitarios sigue siendo insuficiente. A pesar de la creciente literatura sobre desastres, la investigación sobre el trauma posterior a un terremoto y las estrategias de afrontamiento entre los estudiantes de ciencias de la salud en Turquía sigue siendo limitada^(10,11)^. Esta brecha es crítica, ya que estos estudiantes representan tanto a personas directamente afectadas como a futuros profesionales de la salud en situaciones de desastre, donde la doble exposición puede estar asociada con un mayor malestar y patrones de afrontamiento. Dada la posibilidad de futuros terremotos en Turquía y el riesgo de que los estudiantes sufran un nuevo trauma, los efectos psicológicos podrían persistir con el tiempo, lo que contribuiría a un trauma, miedo y ansiedad sostenidos, así como a alteraciones en el funcionamiento social y los sistemas de apoyo. Por consiguiente, un análisis simultáneo del trauma y las estrategias de afrontamiento es esencial para captar el estado psicosocial de los estudiantes e identificar tanto las vulnerabilidades como los factores protectores que puedan orientar intervenciones específicas para mejorar la resiliencia y la preparación ante desastres.

Este estudio se basa en el Modelo Transaccional de Estrés y Afrontamiento de Lazarus y Folkman, el cual proporciona un marco integral para comprender cómo las personas responden a los eventos estresantes de la vida^(12)^. Según este modelo, los resultados psicológicos surgen de la interacción dinámica entre los factores estresantes, las evaluaciones cognitivas y las estrategias de afrontamiento. También destaca las dimensiones psicosociales al enfatizar el papel de los recursos ambientales y sociales en el proceso de estrés y afrontamiento. En este contexto, el modelo ofrece un marco útil para examinar la relación entre los factores estresantes relacionados con los terremotos, las estrategias de afrontamiento y los niveles de trauma entre los estudiantes de ciencias de la salud. Dado que Turquía se encuentra en una zona sísmica activa y que el riesgo de terremotos sigue siendo una preocupación importante de salud pública, es de gran importancia comprender la adaptación psicológica posterior a los terremotos entre los estudiantes de ciencias de la salud. Estos estudiantes no solo son personas directamente afectadas, sino también futuros profesionales de la salud de quienes se espera que desempeñen un papel clave en la respuesta ante desastres. En consecuencia, este estudio tuvo como objetivo investigar los niveles de trauma y las estrategias de afrontamiento asociadas al estrés sísmico experimentado por los estudiantes de ciencias de la salud tras los terremotos a gran escala que ocurrieron en Turquía en 2023.

### Preguntas de Investigación

(1) ¿Cuáles son los niveles de trauma post-terremoto de los estudiantes de ciencias de la salud?
(2) ¿Qué estrategias de afrontamiento (coping) utilizan los estudiantes de ciencias de la salud?
(3) ¿Existe una relación entre los niveles de trauma y las estrategias de afrontamiento?
(4) ¿Qué factores demográficos y de experiencia están asociados con los niveles de trauma y las estrategias de afrontamiento?

## MÉTODO

### Tipo de Investigación

Este es un estudio transversal.

### Población y Muestra de Investigación

Este estudio se llevó a cabo mediante una encuesta en línea de autoinforme entre el 10 de abril de 2023 y el 25 de julio de 2023. La población objetivo estuvo compuesta por estudiantes matriculados en facultades de ciencias de la salud de universidades de Turquía. Se empleó una estrategia de muestreo no probabilístico, que incluyó métodos de muestreo por conveniencia y en bola de nieve. Se utilizó un software estadístico de acceso público (OpenEpi v.3) para estimar el tamaño mínimo de muestra requerido, el cual se calculó en 385 participantes con base en un nivel de significación de 0,05 y un intervalo de confianza del 95%^(13)^. Participaron en el estudio un total de 398 estudiantes, lo que superó el tamaño mínimo requerido de la muestra y, por lo tanto, garantizó una potencia estadística suficiente para los análisis. Los participantes fueron reclutados a través de profesores y grupos de estudiantes en redes sociales. Debido a que se utilizaron el muestreo por conveniencia y el muestreo en bola de nieve, y a que el enlace de la encuesta se difundió a través de múltiples canales, no se pudo determinar el número total de estudiantes que recibieron la invitación; por lo tanto, no se pudo calcular una tasa de respuesta.

### Criterios de Selección

Los criterios de inclusión fueron: (1) tener 18 años o más, (2) ser estudiante de un programa de ciencias de la salud (la elegibilidad se determinó mediante una pregunta de selección de autoinforme en la que se preguntaba si los participantes estaban inscritos en un programa de ciencias de la salud), (3) aceptar participar voluntariamente y (4) tener acceso a Internet. No se incluyó a las personas que informaron tener afecciones graves de salud física o mental que pudieran impedir la finalización de la encuesta. Los criterios de exclusión fueron: (1) discapacidades auditivas o visuales autoinformadas que pudieran interferir con la participación y (2) retiro del estudio en cualquier etapa.

### Recolección de Datos

La encuesta incluyó preguntas sobre las características de los participantes, así como dos escalas previamente desarrolladas y validadas: la Escala de Determinación del Nivel de Trauma Posterior al Terremoto (PETLDS)^(14)^ y la Escala de Afrontamiento del Estrés por Terremoto (CESS)^(15)^. Una vez que el Comité de Ética de la Universidad de Amasya otorgó la autorización ética, se invitó a todos los estudiantes matriculados en programas de pregrado de ciencias de la salud en todas las universidades de Turquía a participar en el estudio y completar la encuesta en línea anónima diseñada con Google Forms. El enlace de la encuesta se distribuyó inicialmente a los profesores y representantes estudiantiles, a quienes se les pidió que lo reenviaran a los estudiantes elegibles a través de los canales de comunicación institucionales y los grupos de redes sociales de los estudiantes. También se animó a los participantes a compartir la encuesta con otros estudiantes elegibles de ciencias de la salud. La configuración de Google Forms se ajustó para restringir los envíos múltiples desde una misma cuenta.

Los estudiantes que aceptaron participar en el estudio vieron la hoja informativa y el formulario de consentimiento como primera página al hacer clic en el enlace; tras solicitarse que leyeran detenidamente la hoja informativa y el formulario de consentimiento, se les pidió que firmaran electrónicamente su aprobación del formulario, tras lo cual tuvieron acceso en línea a los formularios de recolección de datos en forma de encuesta. La encuesta en línea se diseñó para garantizar el anonimato de los participantes al no recolectar ninguna información de identificación personal. Los datos se almacenaron en una plataforma segura, protegida con contraseña, a la que solo tenían acceso los investigadores. Todas las respuestas fueron confidenciales y la participación fue voluntaria. Estas medidas cumplieron con las directrices éticas para la investigación que involucra datos sensibles relacionados con traumas, con el fin de proteger la privacidad de los participantes y fomentar respuestas correctas. El enlace de la encuesta se difundió entre los estudiantes a través de los profesores de los cursos, quienes lo reenviaron a sus alumnos por correo electrónico. La encuesta se dio a conocer en los grupos de redes sociales (Facebook, Instagram y WhatsApp) de los estudiantes de ciencias de la salud. Se enviaron recordatorios periódicos a los profesores y se compartieron publicaciones periódicas en grupos populares de redes sociales para alentar a los estudiantes a completar la encuesta cada dos semanas. El tiempo promedio para completar la encuesta fue de aproximadamente 10 a 15 minutos.

### Herramientas de Recolección de Datos

***Formulario de información del participante (PIF):*** Este formulario fue creado por los investigadores de acuerdo con la literatura^(14,16)^. Las preguntas sobre las características de los participantes incluían la edad, el género, el departamento de ciencias de la salud, la ciudad de residencia, la experiencia del terremoto del 6 de febrero, el efecto del terremoto en la salud física y mental, y el impacto del terremoto en la educación.

***Escala de Determinación del Nivel de Trauma Posterior al Terremoto (Post-Earthquake Trauma Level Determination Scale – PETLDS):*** La PETLDS fue desarrollada en turco y validada en 2013 por Tanhan y Kayri^(14)^ para medir el nivel de trauma después de un terremoto. Consta de 20 ítems agrupados en cinco subescalas: (1) estructura cognitiva, (2) problemas conductuales, (3) conductas afectivas, (4) limitación emocional y (5) problemas para dormir. Cada pregunta se evalúa mediante una escala tipo Likert de cinco puntos (del 1 al 5, donde 1 significa “para nada” y 5 significa “siempre”), con puntuaciones totales que oscilan entre 20 y 100. Una puntuación más alta indica un mayor nivel de trauma después de un terremoto. Una puntuación de 52,385 ± 5,051 según la escala indica un valor umbral a partir del cual las personas se consideran traumatizadas^(14)^. Los valores por encima y por debajo de este umbral indican niveles bajos y altos de traumatización, respectivamente. El PETLDS es un instrumento turco conocido por su sólida validez científica, con un alfa de Cronbach de 0,87, lo que demuestra una buena consistencia interna^(14)^.

***Escala de Afrontamiento (coping) del Estrés por Terremoto* (Coping with Earthquake Stress Scale *CESS*):** La CESS fue desarrollada en turco y validada por Yondem y Altay^(15)^. La escala de 16 ítems mide las emociones, los pensamientos y los comportamientos utilizados para afrontar el estrés por terremoto. Consta de tres subescalas: (1) afrontamiento religioso (preguntas 2, 3, 9, 10 y 11), (2) reevaluación positiva (preguntas 7, 12, 13, 14, 15 y 16), y la búsqueda de (3) apoyo social (preguntas 1, 3, 4, 6 y 7). Cada pregunta se mide mediante la escala de Likert de cuatro puntos (del 1 al 4, donde 1 significa “para nada” y 4 significa “siempre”) y se califica del 1 al 4. La escala no arroja una puntuación total, ya que cada subescala se califica por separado. Las puntuaciones totales oscilaron entre 5 y 20 para la afrontamiento religioso, entre 6 y 24 para la reevaluación positiva y entre 5 y 20 para la búsqueda de apoyo social. Las puntuaciones más altas indican un mayor uso de esta estrategia de afrontamiento^(15)^. Se determinó que los coeficientes alfa de Cronbach para la CESS fueron α = 0,85 para el afrontamiento religioso, α = 0,69 para la reevaluación positiva y α = 0,74 para la búsqueda de apoyo social^(15)^.

### Aspectos Éticos

Se informó a todos los participantes sobre los detalles del estudio y estos aceptaron participar en la investigación. El estudio fue aprobado por el Comité de Ética de la Universidad de Amasya (Fecha: 10 de abril de 2023; N.º de aprobación: E-76988455-050.01.04-125195). Se obtuvo la aprobación ética antes de la recolección de datos. Se invitó a participar a todos los estudiantes matriculados en programas de pregrado de ciencias de la salud en universidades de Turquía. La primera página de la encuesta en línea (Google Forms) incluía una hoja informativa y un formulario de consentimiento electrónico. Se requirió a los participantes que leyeran la hoja informativa y dieran su consentimiento informado electrónicamente antes de acceder al cuestionario. Quienes dieron su consentimiento participaron en el estudio y completaron la encuesta. El estudio se llevó a cabo de conformidad con la Declaración de Helsinki. No se utilizó inteligencia artificial en ninguna parte de este estudio.

### Análisis de Datos

Los datos se ingresaron y analizaron utilizando el paquete estadístico SPSS para Windows 24.0 (IBM Inc, Armonk, NY, EE. UU.). Para presentar los datos se utilizaron el mínimo, el máximo, la media, la desviación estándar, la frecuencia y el porcentaje. Se emplearon estadísticas descriptivas (media, desviación estándar [DE] o número, frecuencia) para presentar los datos. Se utilizó la prueba de Kolmogorov-Smirnov para evaluar la normalidad de los datos antes de seleccionar las pruebas, lo cual reveló que los datos seguían una distribución normal. Se utilizaron el coeficiente de correlación de Pearson (para las relaciones entre PETLDS, CESS y la edad) y el coeficiente de correlación de Spearman (para las relaciones con otras variables categóricas) a fin de examinar las relaciones entre los niveles de trauma y las estrategias de afrontamiento. Estos análisis de correlación son métodos adecuados para investigar las relaciones entre variables. Se reportaron los valores de Rho para mostrar la fuerza de la asociación. Se realizaron análisis de regresión lineal múltiple para identificar los factores que podrían predecir los niveles de trauma y las estrategias de afrontamiento. Dado que el estudio se basó en datos de encuestas de autoinforme, se llevó a cabo la prueba de un solo factor de Harman para evaluar el riesgo de varianza de método común (CMV). Se realizó un análisis factorial exploratorio (AFE) sin rotación utilizando todos los ítems de las escalas de medición. Todos los participantes respondieron todas las preguntas; no hubo datos faltantes. Los resultados se evaluaron con un intervalo de confianza del 95% y un nivel de significación de p < 0,05.

## RESULTADOS

Un total de 398 estudiantes completaron la encuesta. La edad promedio de los participantes fue de 20,9 ± 1,7 años (rango: 18–30 años). La mayoría de los estudiantes eran mujeres (82%) y estudiaban enfermería (55%). Un tercio de los estudiantes (33%) informó haber vivido el terremoto. Muchos de los estudiantes (39%) estaban cursando estudios a distancia. La mitad de los estudiantes (50%) informó que el terremoto había tenido un efecto negativo en sus vidas (Tabla 1).

**Tabla 1:** Características de los participantes (N = 398) – TR, Turquía, 2023.

Variables	Number (%)
**Sexo**	
Mujeres	325 (82)
Hombres	73 (18)
**Departamento**	
Enfermería	218 (55)
Obstetricia	81 (20)
Nutrición y dietética	53 (13)
Fisioterapia	46 (12)
**Experiencia del terremoto**	
Sí	133 (33)
No	265 (67)
**Existencia de algún problema de salud a causa del terremoto**	
Sí	56 (14)
No	342 (86)
**Estado de salud física tras el terremoto**	
Muy bueno	6 (2)
Bueno	24 (6)
Aceptable	233 (59)
Deficiente	101 (25)
Muy malo	34 (8)
**Estado de salud psicológica tras el terremoto**	
Muy bueno	0
Bueno	1 (1)
Aceptable	26 (6)
Deficiente	184 (46)
Muy malo	187 (47)
**Situación actual de la educación**	
Enseñanza a distancia	156 (39)
Presencial	98 (25)
Híbrido	144 (36)
**Nivel educativo previsto**	
A distancia	113 (29)
Presencial	163 (41)
Híbrido	71 (17)
En pausa	51 (13)
**Repercusiones del terremoto en la vida**	
Muy positivo	3 (1)
Positivo	3 (1)
Ni positivo ni negativo	99 (25)
Negativo	199 (50)
Muy negativo	94 (23)
**Frecuencia con la que se siguen las noticias sobre terremotos**	
Siempre	109 (27)
Muy a menudo	204 (51)
A veces	63 (16)
Rara vez	15 (4)
Nunca	7 (2)

### Niveles de Trauma Tras el Terremoto y Estrategias de Afrontamiento

La puntuación media en la escala PETLDS fue de 54,6 ± 16,81, lo que indica que los estudiantes sufrían un estado de trauma. Entre las distintas especialidades, los estudiantes de enfermería presentaban un menor nivel de trauma que los de otras especialidades. Del mismo modo, los niveles de afrontamiento del estrés posterremoto de los estudiantes de enfermería fueron los más altos (Tabla 2).

**Tabla 2:** Valor medio de los niveles de trauma y estrategias de afrontamiento del estrés posterremoto por departamento – TR, Turquía, 2023.

Departamento	N
		Estrategias para afrontar el estrés posterremoto		Nivel de trauma posterremoto (rango: 20–100)					
				Afrontamiento religioso (rango: 5–20)		Reevaluación positive (rango: 6–24)		Búsqueda de apoyo social (rango: 5–20)	
		mean	DE/EE	media	DE/EE	media	DE/EE	media	DE/EE
Enfermería	218	51,6	1,12	14,8	0,23	17,1	0,22	13,9	0,13
Obstetricia	81	56,7	1,8	13,9	0,38	16,3	0,38	12,9	0,23
Nutrición y dietética	53	61,9	1,97	13,5	0,51	15,1	0,51	12,3	0,3
Fisioterapia	46	56,6	2,69	13,6	0,54	15,3	0,46	12,6	0,33
Total	398	54,6	16,8	14,5	3,55	16,5	3,49	13,1	2,12

N: número; DE: desviación estándar; EE: error estándar.

### Relación Entre la Escala Petlds y Los Factores del CESS

Los resultados del análisis de correlación entre el PETLDS y los niveles de afrontamiento revelaron que los niveles de trauma posterremoto se asociaban con el nivel de afrontamiento mediante reevaluación positiva (r = -0,186; p < 0,001). A medida que aumentaban los niveles de trauma posterremoto de los estudiantes, disminuían los niveles de afrontamiento del estrés posterremoto mediante reevaluación positiva (Tabla 3).

**Tabla 3:** Asociaciones estadísticamente significativas entre los niveles de trauma tras el terremoto, las estrategias de afrontamiento del estrés por el terremoto y otros factores – TR, Turquía, 2023.

	1	2	3	4	5	6	7	8	9	10
Género	–									
Departamento	.120^*^	–								
Experiencia del terremoto	.091	.058	–							
Existencia de problemas de salud derivados del terremoto	.107^*^	.066	.188^***^	–						
Efecto del terremoto en la educación	.062	.015	.114^*^	.150^***^	–					
Frecuencia con la que se siguen las noticias sobre terremotos	.086	.086	.030	0,047	0,136^***^	–				
PETL	.160^***^	0,197^***^	.141^***^	.141^***^	.199^***^	.208^***^	–			
Afrontamiento religioso	.059	.110^*^	0,038	0,037	.163^**^	0,062	0,098	–		
Reevaluación positiva	0,087	0,224^***^	0,018	0,016	0,110^*^	0,018	0,186^***^	.439^***^	–	
Búsqueda de apoyo social	.064	.134^**^	.054	.004	0,049	0,137^**^	0,045	0,278^***^	0,464^***^	–

La tabla presenta los valores de r/rho.

*p < 0,05;

**p < 0,01;

***p < 0,001.

PETL: Nivel de trauma posterremoto.

1. Género; 2. Departamento; 3. Experiencia del terremoto; 4. Existencia de problemas de salud derivados del terremoto; 5. Efecto del terremoto en la educación; 6. Frecuencia con la que se siguen las noticias sobre el terremoto; 7. PETL; 8. Afrontamiento religioso; 9. Reevaluación positiva; 10. Búsqueda de apoyo social.

### Factores Asociados con el PETLDS y el CESS

Las puntuaciones del PETLDS mostraron asociaciones significativas con varios factores demográficos y experienciales. Las estudiantes del sexo femenino declararon niveles de trauma más elevados que los estudiantes del sexo masculino. También se observaron diferencias entre departamentos: las estudiantes de enfermería obtuvieron puntuaciones más bajas en el PETLDS que las estudiantes de otras disciplinas de ciencias de la salud (obstetricia, nutrición y dietética, fisioterapia). Además, los estudiantes que vivieron directamente el terremoto, ya fuera por encontrarse en la zona afectada o por exposición física o emocional, obtuvieron puntuaciones en el PETLDS significativamente más altas que aquellos que no se vieron directamente afectados. Del mismo modo, los estudiantes que experimentaron problemas de salud relacionados con el terremoto (como lesiones o enfermedades) también informaron de niveles elevados de trauma. También se observó una asociación positiva entre las puntuaciones en la escala PETLDS y la percepción de los estudiantes de que el terremoto pudiera afectar a su educación. Aquellos que consideraban que la catástrofe había alterado su vida académica eran más propensos a informar de niveles de trauma más elevados. Además, una mayor frecuencia en el seguimiento de las noticias relacionadas con el terremoto se asoció con mayores niveles de trauma (Tabla 3).

La afrontamiento religioso ante el estrés provocado por el terremoto se asoció con la especialidad de estudios (enfermería) y con el efecto del terremoto en la educación. Los estudiantes de enfermería presentaron niveles más elevados de afrontamiento religioso (r = 0,110; p = 0,028). Se observó una débil correlación negativa entre la afrontamiento religioso y el posible efecto del terremoto en la educación (r = −0,163, p = 0,001); quienes pensaban que el terremoto afectaría a la educación presentaban niveles más bajos de afrontamiento religioso. Del mismo modo, la reevaluación positiva como estrategia de afrontamiento del estrés provocado por el terremoto también se asoció con el departamento de estudios y el efecto del terremoto en la educación. Los estudiantes de enfermería presentaban niveles más altos de reevaluación positiva (r = 0,224; p < 0,001), y se observó una correlación negativa débil entre la reevaluación positiva como estrategia de afrontamiento y el efecto del terremoto en la educación (r = −0,110; p = 0,028) (Tabla 3).

Se observó que los niveles de afrontamiento mediante la búsqueda de apoyo social estaban asociados con la pertenencia de los estudiantes a un departamento concreto, y que los estudiantes de enfermería presentaban niveles más altos que los de otros departamentos (r = 0,134; p = 0,007). También se observó una asociación con la frecuencia con la que se seguían las noticias sobre el terremoto. Aquellos que seguían las noticias con mayor frecuencia presentaban niveles más bajos de búsqueda de apoyo social para afrontar el estrés del terremoto (r = −0,137, p = 0,006) (Tabla 3).

Se llevó a cabo un análisis de regresión lineal múltiple para examinar si las variables demográficas y relacionadas con el sismo permitían predecir los niveles de trauma. Las variables independientes fueron la edad, el género, el departamento, el año de estudio, la experiencia del sismo, la frecuencia con la que se seguían las noticias sobre el sismo y el efecto del sismo en la educación. Entre los predictores, el género resultó ser un predictor significativo, ya que las mujeres reportaron niveles de trauma más altos que los hombres (β = −0,153, p = 0,002). El departamento se asoció positivamente con las puntuaciones de trauma (β = 0,156, p = 0,001), lo que indica diferencias entre los programas académicos. El año de estudio fue un predictor negativo (β = −0,128, p = 0,025), lo que sugiere que los estudiantes en años más avanzados experimentaron niveles de trauma más bajos. Haber vivido un terremoto también se asoció con niveles más altos de trauma (β = −0,152, p = 0,002), al igual que la frecuencia con la que se seguían las noticias relacionadas con el terremoto (β = −0,177, p < 0,001). La edad se acercó a la significación (β = 0,106, p = 0,060), pero no fue estadísticamente significativa, y el efecto percibido del terremoto en la educación no fue un predictor significativo (β = −0,013, p = 0,785).

Se llevó a cabo un análisis de regresión múltiple para examinar si las variables demográficas y relacionadas con el terremoto predecían las estrategias de afrontamiento. Entre los predictores, el departamento fue un predictor estadísticamente significativo para el afrontamiento religioso (β = −0,133, p = 0,008), la reevaluación positiva (β = −0,222, p < 0,001) y la búsqueda de apoyo social (β = −0,157, p = 0,002), lo que indica que los estudiantes del departamento de enfermería reportaron puntuaciones más altas en estas estrategias de afrontamiento en comparación con los estudiantes de otros departamentos. Además, la frecuencia con la que se seguían las noticias relacionadas con el sismo predijo de manera significativa la búsqueda de apoyo social (β = −0,144, p = 0,004), lo que sugiere que los estudiantes que seguían las noticias con mayor frecuencia reportaron niveles más bajos de búsqueda de apoyo social.

## DISCUSIÓN

Los terremotos de febrero de 2023 en Turquía, descritos como el “*desastre del siglo*”, se asociaron con importantes desafíos físicos, psicológicos y psicosociales. El objetivo de este estudio fue investigar los niveles de trauma y las estrategias de afrontamiento asociadas al estrés por el terremoto que experimentaron los estudiantes de ciencias de la salud tras los terremotos a gran escala que ocurrieron en Turquía en 2023.

### Trauma Posterremoto

Los desastres naturales, como los terremotos, son uno de los eventos importantes que causan trauma^(16)^ y tienen un impacto significativo en la salud mental de los sobrevivientes^(8,17)^. Se ha demostrado que los niveles de trauma posterremoto están asociados con una variedad de problemas de salud mental y física en la gran mayoría de la población general^(16,18)^. En este estudio, la puntuación media en la escala PETLDS fue de 54,6 ± 16,81. Tanhan y Kayri^(14)^ señalan que las puntuaciones superiores al valor de corte de 52,385 ± 5,051 indican que los estudiantes se encuentran en estado de trauma. Este valor fue de 23,12 ± 15,03 en el estudio de Kardas y Tanhan^(16)^ con estudiantes universitarios que habían vivido el terremoto de Van en Turquía. En un estudio de Çakır et al.^(19)^, que examinó los niveles de trauma y las estrategias de afrontamiento del estrés posterremoto entre estudiantes universitarios que hacían ejercicio y aquellos que no lo hacían después de un terremoto, el valor fue de 70,94. Estas diferencias pueden estar relacionadas con variaciones en la gravedad y el alcance de los desastres, el nivel de exposición directa y el momento de la recolección de datos en relación con el evento. Específicamente, las diferencias en las características de la muestra, como la edad, el género, el nivel socioeconómico y si los participantes estuvieron expuestos directa o indirectamente al terremoto, pueden haber influido en los niveles de trauma reportados. Además, el tipo de herramientas de medición y los métodos de recolección de datos utilizados podrían haber afectado los resultados. El contexto cultural también pudo haber desempeñado un papel importante, ya que el procesamiento colectivo del trauma, los sistemas de apoyo social y el acceso a los servicios psicosociales varían entre las distintas regiones y comunidades.

En un estudio realizado por Yokoyama et al.^(20)^, que examinó la salud mental tras el terremoto y el tsunami del este de Japón del 11 de marzo de 2011, se observó una alta prevalencia de problemas de salud mental entre los sobrevivientes. De manera similar, una revisión sistemática y un metaanálisis sobre el trastorno de estrés postraumático destacaron la necesidad de que los gobiernos locales implementen intervenciones psicológicas efectivas para las personas afectadas por los terremotos^(17)^. Las investigaciones también indican que las respuestas de las personas ante el trauma están asociadas con sus mecanismos de afrontamiento^(6,19)^. Por lo tanto, fortalecer la capacidad de afrontamiento requiere abordar de manera inmediata e integral las necesidades básicas, tales como la vivienda, la nutrición, la educación y la seguridad tras los desastres. Esto es especialmente crucial para los estudiantes universitarios cuya educación se ve interrumpida, no solo por un cambio repentino a la educación a distancia, sino también por la imposibilidad de completar la capacitación práctica esencial en entornos clínicos o de laboratorio. La implementación de intervenciones culturalmente sensibles que respeten los valores sociales y los estilos colectivos de afrontamiento puede brindar apoyo psicológico específico y ayudar a restablecer tanto la continuidad académica como el bienestar general.

### Factores Asociados con el PETLDS y el CESS de los Estudiantes

En el estudio, se observaron niveles más altos de trauma posterior al terremoto entre las estudiantes mujeres, quienes vivieron directamente el terremoto, quienes reportaron problemas de salud, quienes percibieron que su educación se vio afectada y quienes estuvieron expuestos con frecuencia a noticias relacionadas con el terremoto. Estas asociaciones pueden reflejar la coexistencia de múltiples factores estresantes, como la pérdida de seres queridos, los daños materiales y la interrupción de la vida cotidiana y la educación. La exposición continua a los medios de comunicación pudo haber estado relacionada con una mayor excitación emocional y una angustia psicológica sostenida a lo largo del tiempo, lo que podría haber intensificado los recuerdos traumáticos. Además, el cambio abrupto a la educación en línea pudo haber estado asociado con una mayor interrupción académica, un menor apoyo social y una mayor incertidumbre. Cabe destacar que las estudiantes de enfermería reportaron niveles más bajos de trauma y mayores habilidades de afrontamiento en todas las subdimensiones del CESS. Una posible explicación es que los estudiantes de enfermería puedan beneficiarse de la exposición clínica temprana y de la capacitación relacionada con el manejo del estrés y la preparación para desastres. Sin embargo, estos factores no se midieron directamente en este estudio y, por lo tanto, deben interpretarse con cautela. Además, estas diferencias también pueden verse influenciadas por la autoselección en el programa de enfermería, así como por posibles variaciones en las características demográficas, como el género, la distribución y la edad, y la exposición previa a experiencias relacionadas con la atención de la salud o estresantes en diversas disciplinas académicas. No obstante, la mayoría de las asociaciones observadas fueron de baja magnitud. Aunque estadísticamente significativas, estas hallazgos pueden tener una importancia práctica limitada y deben interpretarse con cautela. Otros factores contextuales, como los sistemas de apoyo institucional, las condiciones socioeconómicas y el acceso a servicios de salud mental, también pueden ser relevantes para explicar las diferencias entre los grupos de estudiantes.

De acuerdo con los presentes resultados, estudios previos en la literatura han demostrado que los factores de riesgo comunes tras un terremoto son el género femenino^(19,20)^, la edad avanzada^(21)^, el bajo nivel educativo y el estatus socio-económico^(22)^, la exposición al terremoto, las enfermedades psiquiátricas previas^(23)^, el desplazamiento tras el terremoto, las redes sociales deficientes y las pérdidas económicas^(24)^.

### Asociaciones Entre los Niveles de PETLDS de los Estudiantes y la CESS

En el estudio, se observó una relación negativa estadísticamente significativa entre los niveles de trauma posterior al terremoto y la capacidad de los estudiantes para afrontar el estrés del terremoto mediante la reevaluación positiva. La reevaluación positiva, un proceso cognitivo que implica replantear un evento estresante para encontrar crecimiento personal o significado, está relacionada con un mayor bienestar psicológico y se considera un mecanismo de afrontamiento centrado en el problema^(15)^. Es un elemento central de las terapias cognitivo-conductuales para la depresión y el manejo del estrés, lo que resalta su papel en la afrontamiento adaptativo y la resiliencia^(25,26)^. La asociación negativa observada sugiere que los estudiantes que informaron un mayor uso de la reevaluación positiva también tendieron a reportar niveles más bajos de trauma. Sin embargo, la naturaleza transversal del estudio no permite sacar conclusiones sobre si la reevaluación positiva reduce los síntomas de trauma o si un menor nivel de trauma facilita el uso de la reevaluación positiva. Este hallazgo también concuerda con el Modelo Transaccional de Estrés y Afrontamiento de Lazarus y Folkman, en el que reformular los factores estresantes es una técnica de afrontamiento centrada en las emociones que fortalece la resiliencia y reduce la amenaza percibida^(12)^. La reevaluación positiva puede ayudar a las personas a sentirse más en control y a afrontar mejor las situaciones, lo que puede protegerlas del impacto psicológico de los eventos traumáticos. Los estudiantes de enfermería de este estudio reportaron niveles más altos de reevaluación positiva, junto con puntuaciones más bajas de trauma, lo que respalda aún más la teoría de que unos mecanismos internos de afrontamiento más sólidos se asocian con un impacto reducido del trauma. Por el contrario, los niveles más altos de trauma pueden estar asociados con un uso reducido de dichas estrategias cognitivas adaptativas, que son esenciales para la resiliencia psicológica. Estos hallazgos sugieren que las intervenciones psicosociales y los programas de capacitación destinados a fortalecer las estrategias de afrontamiento adaptativas merecen una investigación más profunda, aunque su efectividad no puede inferirse a partir del presente estudio.

Además, se encontró una asociación positiva entre la percepción de los estudiantes de que el terremoto afectó su vida educativa y tanto la reevaluación positiva como la afrontamiento religioso. Esta asociación podría indicar que los estudiantes que experimentaron una mayor interrupción educativa eran más propensos a reportar el uso de estrategias de reevaluación positiva y de afrontamiento religioso. En algunos estudios, se ha encontrado que las personas recurren a métodos de afrontamiento religioso positivo después de los terremotos^(19,27)^. La afrontamiento religioso, como estrategia centrada en las emociones, puede brindar sentido y consuelo, y puede facilitar la reevaluación positiva al ayudar a las personas a reinterpretar los eventos adversos de una manera más constructiva. La reevaluación positiva, a su vez, refleja un esfuerzo cognitivo por reconstruir las experiencias estresantes de una manera que mejore el control percibido y la estabilidad emocional. Algunos estudios indican que la afrontamiento religioso es una forma común y eficaz de manejar el estrés durante eventos traumáticos como los terremotos; del mismo modo, se ha demostrado que ver la adversidad desde un marco espiritual y buscar fortaleza a través de la fe alivia la angustia y favorece el bienestar psicológico^(15,25)^. En contextos culturalmente religiosos como Turquía, los desastres suelen interpretarse desde un marco espiritual, y la oración y otras prácticas religiosas sirven como recursos clave de afrontamiento que brindan consuelo y resiliencia^(8,28)^.

La espiritualidad y la religión desempeñan un papel significativo en la gestión del estrés y la adaptación^(29,30)^, y se ha demostrado que estos valores mejoran el bienestar psicológico postraumático^(31)^. El hallazgo del presente estudio de que la estrategia de afrontamiento religiosa fue la más utilizada entre los estudiantes concuerda con esta evidencia y subraya la interacción entre los procesos individuales de afrontamiento y los sistemas de valores culturales más amplios^(32)^. Además, los entornos educativos, que son fundamentales para el desarrollo social y psicológico de los estudiantes, pueden influir aún más en la adopción de estas estrategias de afrontamiento al afectar la forma en que los estudiantes manejan sus responsabilidades académicas junto con la carga emocional de la exposición a desastres. Por otra parte, los hallazgos relacionados con la búsqueda de apoyo social indican que las respuestas de afrontamiento al estrés relacionado con desastres están determinadas tanto por el contexto académico como por la exposición a la información. Específicamente, los estudiantes del departamento de enfermería reportaron puntuaciones más altas en la búsqueda de apoyo social que los estudiantes de otros departamentos, lo que sugiere que las diferencias disciplinarias podrían estar asociadas con variaciones en los comportamientos de búsqueda de apoyo. En este contexto, la búsqueda de apoyo social refleja un esfuerzo activo de afrontamiento dirigido a obtener asistencia emocional e instrumental tras un desastre. Esta interpretación se ve respaldada en parte por investigaciones previas que demuestran que un mayor apoyo social percibido se asocia con un afrontamiento más eficaz entre los estudiantes de enfermería^(33)^, lo que resalta el papel funcional de los recursos interpersonales en la regulación del estrés. Por el contrario, una exposición más frecuente a noticias relacionadas con el terremoto se asoció con puntuaciones más bajas en esta subescala. Esta asociación podría reflejar una menor búsqueda de apoyo entre los estudiantes que informaron una mayor exposición a los medios de comunicación, aunque no se puede determinar la dirección de esta relación. En general, estos hallazgos enfatizan que las intervenciones efectivas posteriores a un desastre dirigidas a los estudiantes deben adoptar un marco integrado que combine enfoques psicológicos basados en la evidencia con perspectivas cultural y espiritualmente sensibles para fortalecer la resiliencia y apoyar la recuperación a largo plazo.

### Fortalezas y Limitaciones del Estudio

Este estudio presenta varias fortalezas y limitaciones. Hasta donde sabemos, es el primer estudio que examina los niveles de trauma y las estrategias de afrontamiento tras el terremoto entre estudiantes de ciencias de la salud en Turquía a raíz de los terremotos de 2023. Aporta evidencia preliminar que puede servir de base para futuras investigaciones sobre las respuestas al trauma y los mecanismos de afrontamiento en poblaciones expuestas a desastres, particularmente en el ámbito de la educación en ciencias de la salud. Aunque existen estudios similares en otras poblaciones universitarias, la evidencia que se centra específicamente en los estudiantes de ciencias de la salud sigue siendo limitada, lo que refuerza la contribución de este estudio. Los hallazgos también resaltan la importancia de integrar la capacitación en manejo del estrés y estrategias de afrontamiento en los planes de estudio de las ciencias de la salud para preparar mejor a los estudiantes para futuros desafíos psicológicos relacionados con desastres. En general, los resultados subrayan la necesidad de una educación en ciencias de la salud centrada en la resiliencia e informada sobre el trauma, con implicaciones importantes para los planes de estudio de enfermería y educación para la salud.

A pesar de estas fortalezas, deben reconocerse varias limitaciones. En primer lugar, se utilizó un enfoque de muestreo no probabilístico (muestreo por conveniencia y en bola de nieve a través de plataformas en línea y redes universitarias), lo que pudo haber introducido un sesgo de selección y limita la representatividad de la muestra. En consecuencia, los hallazgos deben interpretarse con cautela y es posible que no sean generalizables a todos los estudiantes de ciencias de la salud en Turquía. Aunque la muestra fue adecuada para los análisis estadísticos, los participantes fueron reclutados de múltiples universidades sin registrar sistemáticamente la distribución institucional, y la muestra presentaba un desequilibrio en cuanto al género y el campo de estudio, lo que pudo haber afectado la representatividad.

En segundo lugar, el diseño transversal impide sacar conclusiones sobre relaciones causales. Por lo tanto, las asociaciones observadas entre los niveles de trauma, las estrategias de afrontamiento y las características de los participantes no deben interpretarse como evidencia de relaciones de causa y efecto. Además, una parte de los participantes había vivido el terremoto de manera indirecta, lo que pudo haber influido en los niveles de trauma reportados y debe tenerse en cuenta al interpretar los resultados. El estudio tampoco evaluó variables potencialmente importantes, como los antecedentes psiquiátricos previos, las experiencias traumáticas previas, la autoeficacia, el apoyo social percibido y el contexto sociocultural, todos los cuales pudieron haber influido en las respuestas al trauma y las estrategias de afrontamiento. Por último, el uso de medidas autoinformadas pudo haber introducido sesgos de respuesta y de deseabilidad social. Se necesitan futuros estudios longitudinales que utilicen métodos de muestreo basados en la probabilidad para confirmar estos hallazgos y proporcionar una comprensión más integral de los niveles de trauma y los procesos de afrontamiento entre los estudiantes de ciencias de la salud.

## CONCLUSIÓN

Este estudio ofrece información importante sobre los niveles de trauma y las estrategias de afrontamiento de los estudiantes universitarios tras los terremotos ocurridos el 6 y el 20 de febrero de 2023. Los hallazgos indican que, en general, los niveles de trauma posteriores al sismo entre los estudiantes de ciencias de la salud fueron elevados. Los niveles de trauma se asociaron significativamente con varios factores, entre ellos el género, el departamento académico, la percepción de la interrupción de la vida académica, la exposición directa al sismo, los problemas de salud relacionados con el sismo y la frecuencia con la que se seguían las noticias relacionadas con el sismo. Además, los estudiantes de enfermería mostraron niveles más bajos de trauma y mayores capacidades de afrontamiento en comparación con los estudiantes de otros departamentos.

Un análisis más detallado reveló que los estudiantes de enfermería informaron un mayor uso de estrategias de afrontamiento religiosas, reevaluación positiva y búsqueda de apoyo social, al tiempo que experimentaban niveles más bajos de trauma. Estos hallazgos concuerdan con el Modelo Transaccional de Estrés y Afrontamiento de Lazarus y Folkman, lo que respalda el papel de los procesos de evaluación cognitiva y las estrategias de afrontamiento en la configuración de las respuestas psicológicas de las personas ante eventos traumáticos.

En general, los resultados resaltan la necesidad de contar con sistemas integrales de apoyo emocional, social y psicológico para los estudiantes de ciencias de la salud, especialmente considerando su futuro papel como profesionales de la salud de primera línea. Estos resultados indican que las estrategias de afrontamiento adaptativas, como la reevaluación positiva, el afrontamiento religioso y el uso del apoyo social, así como la inclusión de habilidades de afrontamiento y capacitación en preparación para desastres en los planes de estudio de ciencias de la salud, pueden apoyar la adaptación psicológica de los estudiantes tras los desastres. El estudio también subraya la importancia de realizar más investigaciones para profundizar en la comprensión de los procesos traumáticos y los mecanismos de afrontamiento posteriores a un desastre.

## DISPONIBILIDAD DE DATOS

El conjunto completo de datos que respalda los resultados de este estudio se publicó en el propio artículo.
